# Supplementary material for: Habitat selection by wolves and mountain lions during summer in western Montana
Source: PLoS One. 2021 Jul 22;16(7):e0254827. doi: 10.1371/journal.pone.0254827 (PMC8297843; doi:10.1371/journal.pone.0254827)
Supplement: Attachment — Submitted filename: Response_to_reviewers.docx [file pone.0254827.s004.docx]

PONE-D-21-03244R1
Habitat selection by wolves and mountain lions during summer in western Montana
PLOS ONE

Dear Dr. Peterson,

Thank you for submitting your manuscript to PLOS ONE. After careful consideration, we feel that it has merit but does not fully meet PLOS ONE’s publication criteria as it currently stands. Therefore, we invite you to submit a revised version of the manuscript that addresses the points raised during the review process.

Please submit your revised manuscript by 28 June 2021. If you will need more time than this to complete your revisions, please reply to this message or contact the journal office at [plosone@plos.org](mailto:plosone@plos.org). When you're ready to submit your revision, log on to <https://www.editorialmanager.com/pone/> and select the 'Submissions Needing Revision' folder to locate your manuscript file.

Please include the following items when submitting your revised manuscript:

- A rebuttal letter that responds to each point raised by the academic editor and reviewer(s). You should upload this letter as a separate file labeled 'Response to Reviewers'.
- A marked-up copy of your manuscript that highlights changes made to the original version. You should upload this as a separate file labeled 'Revised Manuscript with Track Changes'.
- An unmarked version of your revised paper without tracked changes. You should upload this as a separate file labeled 'Manuscript'.

If you would like to make changes to your financial disclosure, please include your updated statement in your cover letter. Guidelines for resubmitting your figure files are available below the reviewer comments at the end of this letter.

If applicable, we recommend that you deposit your laboratory protocols in [protocols.io](http://protocols.io/) to enhance the reproducibility of your results. Protocols.io assigns your protocol its own identifier (DOI) so that it can be cited independently in the future. For instructions see: <http://journals.plos.org/plosone/s/submission-guidelines#loc-laboratory-protocols>. Additionally, PLOS ONE offers an option for publishing peer-reviewed Lab Protocol articles, which describe protocols hosted on [protocols.io](http://protocols.io/). Read more information on sharing protocols at <https://plos.org/protocols?utm_medium=editorial-email&utm_source=authorletters&utm_campaign=protocols>.

We look forward to receiving your revised manuscript.

Kind regards,

W. David Walter, Ph.D.
Academic Editor
PLOS ONE

Journal Requirements:

Please review your reference list to ensure that it is complete and correct. If you have cited papers that have been retracted, please include the rationale for doing so in the manuscript text, or remove these references and replace them with relevant current references. Any changes to the reference list should be mentioned in the rebuttal letter that accompanies your revised manuscript. If you need to cite a retracted article, indicate the article’s retracted status in the References list and also include a citation and full reference for the retraction notice.

Additional Editor Comments (if provided):

I appreciate the authors response to reviewers, specifically on the caveats of using GPS and VHF datasets in your analyses. As I read through both drafts of your manuscript, I shared the concerns of Reviewer #1 that the authors chose to assess resource selection of 2 predators collected with 2 technologies (GPS and VHF) using a single method (3rd order RSF) across numerous study areas. While those methods would be appropriate for the limited dataset collected with VHF technology, there are more appropriate methods for use with GPS technology. Justification for the study design was provided in Lines 227-230 that "...none of these studies assessed resource selection via a used-available design..." It seems that another study could be done in the future using only your GPS collar data from both species and the justification would be similar to the authors by stating "......none of these studies assessed resource selection via a step selection approach..." or similar reasoning. Is there a particular reason the authors chose RSF over more detailed methods using step selection or individual location-specific approaches with only the GPS datasets? Do the authors feel their study design and methods on several study areas is more reliable and beneficial than on a study design using only GPS-collared predators?

CJP: On of the major goals of our study was to assess the generalizability of our predictions of predator space-use to other regions in Montana. Outside of the Garnet Range, the only other mountain lion telemetry data we had available was VHF-sourced. These VHF data had low temporal resolution, therefore, precluding the assessment of step selection function, which require location data sampled at a fine temporal resolution. Therefore, we chose to use RSFs for mountain lions and wolves, and wanted to keep our methodology consistent between predator species. At the end of the introduction, we added:

“To assess the generality of our RSFs, we applied each study area-specific RSF to out-of-sample GPS telemetry data from separate study areas for wolves and assessed their predictive performance. Very high frequency (VHF) telemetry datasets were the only out-of-sample telemetry data available for mountain lions in northwest Montana (20), and consisted of low sample sizes relative to GPS datasets. This precluded us from assessing the generality of more detailed models like step selection functions because they require animal locations sampled at finer temporal scales than standard RSFs (55). However, VHF data enabled us to test the generality of mountain lion RSFs, and we used RSFs for both mountain lions and wolves to keep our methods consistent between species."

We added the reference to Thurfjell et al. (2014) to the reference list to back the need for fine temporal resolution in SSFs.

We added the reference to Krawchuk et al. (2014) to back the claim that wolves can affect mountain lion habitat selection.

Line 154: remove plural for "lion" but add plural for "area".

CJP: Done

Line 158: Change "The Garnets" to the "Garnett Range" which is should be referred to as throughout for consistency (also Line 163).

CJP: Done

Line 160: Remove "Garnet" from mountain lions considering this is mentioned in the paragraph describing the Garnett Range.

CJP: Done

Lines 223-226: The author identify the "MFWP's biomedical protocol for free-ranging mountain lions" and wolves but only report University IACUC #A3327-01 for mountain lions in the Whitefish Range. Were any other Institutional Animal Care and Use Protocols conducted for lions or wolves for any of the other studies?

CJP: We were unable to locate IACUC protocol records for mountain lion data from the Rocky Mountain Front. Detailed capture and handling protocols for mountain lions on the Rocky Mountain Front are outlined in:

Williams, J. S. (1992). *Ecology of mountain lions in the Sun River area of northern Montana by*. Montana State University.

For wolves, I tracked down the IACUC protocol: (AUP # 070–17). I added this to the manuscript.

CJP:

Sawyer H, Nielson RM, Lindzey F, McDonald LL. Winter habitat selection of mule deer before and during development of a natural gas field. J Wildl Manage. 2006;70(2):396–403.

[Note: HTML markup is below. Please do not edit.]


[NOTE: If reviewer comments were submitted as an attachment file, they will be attached to this email and accessible via the submission site. Please log into your account, locate the manuscript record, and check for the action link "View Attachments". If this link does not appear, there are no attachment files.]

While revising your submission, please upload your figure files to the Preflight Analysis and Conversion Engine (PACE) digital diagnostic tool, <https://pacev2.apexcovantage.com/>. PACE helps ensure that figures meet PLOS requirements. To use PACE, you must first register as a user. Registration is free. Then, login and navigate to the UPLOAD tab, where you will find detailed instructions on how to use the tool. If you encounter any issues or have any questions when using PACE, please email PLOS at [figures@plos.org](mailto:figures@plos.org). Please note that Supporting Information files do not need this step.
